# Responses of Low-Quality Soil Microbial Community Structure and Activities to Application of a Mixed Material of Humic Acid, Biochar, and Super Absorbent Polymer

**DOI:** 10.4014/jmb.2003.03047

**Published:** 2020-07-15

**Authors:** Fangze Li, Shuhui Men, Shiwei Zhang, Juan Huang, Xuehua Puyang, Zhenqing Wu, Zhanbin Huang

**Affiliations:** 1 School of Chemical and Environmental Engineering, China University of Mining and Technology, Beijing, 100083, P.R. China; 2 Research Center for Eco-Environmental Sciences, Chinese Academy of Sciences, Beijing, 100085, P.R. China; 3 Shenzhen Techand Ecology and Environment Co., Ltd., Shenzhen, 518040, P.R. China

**Keywords:** Humic acid, biochar, super absorbent polymer, soil improvement, soil microbial community, low-quality soil

## Abstract

Low-quality soil for land reuse is a crucial problem in vegetation quality and especially to waste disposal sites in mining areas. It is necessary to find suitable materials to improve the soil quality and especially to increase soil microbial diversity and activity. In this study, pot experiments were conducted to investigate the effect of a mixed material of humic acid, super absorbent polymer and biochar on low-quality soil indexes and the microbial community response. The indexes included soil physicochemical properties and the corresponding plant growth. The results showed that the mixed material could improve chemical properties and physical structure of soil by increasing the bulk density, porosity, macro aggregate, and promote the mineralization of nutrient elements in soil. The best performance was achieved by adding 3 g·kg^-1^ super absorbent polymer, 3 g·kg^-1^ humic acid, and 10 g·kg^-1^ biochar to soil with plant total nitrogen, dry weight and height increased by 85.18%, 266.41% and 74.06%, respectively. Physicochemical properties caused changes in soil microbial diversity. Acidobacteria, Bacteroidetes, Chloroflexi, Cyanobacteria, Firmicutes, Nitrospirae, Planctomycetes, and Proteobacteria were significantly positively correlated with most of the physical, chemical and plant indicators. Actinobacteria and Armatimonadetes were significantly negatively correlated with most measurement factors. Therefore, this study can contribute to improving the understanding of low-quality soil and how it affects soil microbial functions and sustainability.

## Introduction

Low-quality soil with little human disturbance is used in mine soil reclamation in China, such as in deep tillage and soil replacement operations [1]. However, it has extreme texture with high density, low porosity, low water capacity, and poor fertilizer conservation [2]. The low-quality of guest soil is closely related to soil microbial diversity and activity [3, 4]. In recent years, more attention has been paid to the use of organic materials like humic acid, biochar, and super absorbent polymer for mine soil reclamation.

Humic acid could effectively improve the physical, chemical, and biological properties of soil and promote plant growth. Maji *et al*. [5] found that humic acid could promote plant growth and increase available nutrient content, while dry weight increased 59.49% as compared to control. Super absorbent polymer could increase soil water retention, which could in turn promote fertilizer conservation, improve plant supply capacity, and increase crop yield [6, 7]. Moreover, super absorbent polymers with high water absorption and water retention ability could also promote aggregate formation [8]. Biochar application in soil is beneficial for carbon (C) sequestration, soil fertility, and ecosystem functioning [9, 10]. Lehmann *et al*. [11] and Zhu *et al.* [12] found that the addition of biochar benefited soil microorganisms that play important roles in soil nutrient cycling, carbon exchanges, soil buffering capacity, and soil ecosystem stability. A recent study showed that biochar could improve soil microbial activity, nutrient availability, and plant growth in arid soil [13]. Madhavi *et al.* [14] found that the combined application of humic acid, biochar, and some chemical fertilizers could significantly enhance acid phosphatase activity. Microorganisms regulate several characteristics like soil organic matter, soil nutrient cycle, soil ecosystem stability and soil-buffering capacity, all of which have important effects on the level comprehensive soil fertility [15]. Although there are many studies on the improvement of surface soils with amendments, it is rare to improve the poor soils, such as guest soils for ex-situ remediation and deep tillage for in-situ remediation [16-18]. At the same time, little research has been done on the combination of humic acid, super absorbent polymer and biochar to improve soil and examine the relationship of these materials with the microbial community response [19-21].

In this study, a pot experiment in which the mixed materials of humic acid, biochar, and super absorbent polymer were to soil was conducted to study the effects on low-quality soils such as deep tillage barren soil. The physicochemical properties of soil and microbial community and activities were also investigated to learn the effect of mixed materials on plant growth. This study will enhance low-quality soil improvement and the use for agricultural production and vegetation restoration.

## Materials and Methods

### Site Description

This study was conducted at the Beijing Institute of Light Industry base, in the village of Tingzizhuang, Changping District, Beijing, China (40°19′N, 116°15′E). The area has four distinct seasons with a semi-humid continental monsoon climate. The mean annual sunshine, temperature and precipitation are 2,684 h, 11.8°C, and 550.3 mm, respectively. The physicochemical characteristics of the soil are presented in Table 1.

### Materials

Super absorbent polymer (SAP, powder, consisting of linear anionic polyacrylamide, purity>99%, 60-80 mesh water-soluble materials) was obtained from Beijing Jinyuanyi Ecological Environment Industry Co., Ltd. Humic acid (HA, powder, purity>70%, made by the weathering of lignite) was obtained from Baipo Zhengyuan Fertilizer Co., Ltd., Shijiazhuang, Hebei Province, China. Biochar (B, powder, produced by the pyrolysis of rice husk at 600°C, purity>75%), was obtained from Zhonglian Northwest Engineering Design and Research Institute Co., Ltd, Xi’an, Shanxi Province, China. Rye seeds (winter pasture 70) belonging to *Secale Cereale*, a subspecies of winter rye which is an annual fast-growing variety with long linear leaves and plant heights up to 100 cm.

### Experimental Design

The experiment began in April 2018 in experimental pots (30 cm in diameter and 50 cm in height) containing 10 kg of soil in each pot. A 1 × 1 × 0.6 m square pit was dug for soil collection. The soil was a mixture of half deep soil (5 kg, from the local base soil below 60 cm in depth) and half mature soil (5 kg, from the surface layer of 0- 20 cm) in each treatment. The pot experiments were all performed with a compound fertilizer of 0.1% (w/w) soil weight (N-P-K 18-9-18, Aojia Fertilizer Co., Ltd., China) and 2.5% farm manure (wet weight of cow manure fermentation, moisture content of 28%).

The above soil was used as the base soil and was mixed with super absorbent polymer, humic acid and biochar. The effects of different compositions of the mixed materials were studied as orthogonal test L9(3)^4^ (Table 2), and soil without any treatments was used as a control (CK).

All pots were buried in pits with a depth of 50 cm and a flat bottom. The upper edge of the basin was flat with the ground. They were watered every day to maintain a field water holding capacity of about 60%. After thoroughly mixing the soil and materials for two weeks, twenty rye seeds were sowed into each pot and harvested after 102 d. Each treatment had five replicates (Figs. S1 and S2).

### Determination of Physiochemical Characteristics

Plant height was measured before harvesting. Rye in each pot was harvested, dried at 105°C for 20 min and then dried to constant weight at 80°C. The available nitrogen (N) was determined using the alkali-hydrolysis diffusion method with an elemental analyzer (UV-1100 Spectrophotometer, Germany) [22]. Total N was determined using an SKD-1000 Micro-Kjeldahl Analyzer (Shanghai Peiou Analytical Instrument Co., Ltd). The available phosphorus was measured using the NaHCO_3_ extraction molybdenum-antimony colorimetric method [23]. The soil pH was measured in 10 g soil: 25 mL deionized water using the glass electrode method (NY/T 1377-2007). The soil organic matter was measured using potassium dichromate oxidation titration [24]. The aggregate-size distributions for soil samples were determined by the wet sieving method [25,26]. Soil bulk density was estimated using the ring knife [27]. Specific gravity was determined using the pycnometer method [28]. Porosity was calculated using the following equation [29]:

$$\text{P}=1-\frac{\text{ρ}}{\text{S}}\times 100\;$$

where P is soil porosity (%), ρ is soil bulk density (g·cm^-3^), and S is the soil specific gravity (g·cm^-3^).

### PCR Amplification and Sequencing

Microbial DNA was extracted from soil samples using the E.Z.N.A. soil DNA Kit (Omega Bio-tek, USA). The V3-V4 region of the bacteria 16S ribosomal RNA gene was amplified using PCR (95°C for 2 min, followed by 25 cycles at 95°C for 30 sec, 55°C for 30 sec, and 72°C for 30 sec and a final extension at 72°C for 5 min) with primers 338F (5’-GTA CTC CTA CGG GAG GCA GCA G-3’) and 806R (5’-CCG TCA ATT CMT TTR AGT TT-3’) [30].

The barcode was a unique eight-base sequence for each sample. PCR reactions were performed in triplicate 20 μl mixtures containing 4 μl of 5 × FastPfu Buffer, 2 μl of 2.5 mM dNTPs, 0.8 μl of each primer (5 μM), 0.4 μl of FastPfu Polymerase, and 10 ng of template DNA. Amplicons were extracted from 1.2% agarose gels and purified using the AxyPrep DNA Gel Extraction Kit (Axygen Biosciences, USA) according to the manufacturer’s instructions and quantified using QuantiFluor-ST (Promega, USA). Purified amplicons were pooled, and equimolar samples were paired-end sequenced (2 × 250) on an Illumina MiSeq platform according to the standard protocols. The sequencing data were deposited in the NCBI Sequence Read Archive (SRP187660).

### Data Analysis

Raw fastq files were demultiplexed and quality-filtered using QIIME (version 1.17) with the following criteria: (i) 300 bp reads were truncated at any site receiving an average quality score <20 over a 50 bp sliding window, discarding the truncated reads less than 50 bp; (ii) precise barcode matching, two nucleotide mismatch in primer matching, and reads containing ambiguous characters were removed; (iii) and only sequences that overlap longer than 10 bp were assembled according to their overlap sequence. Reads which could not be assembled were discarded. Operational taxonomical units (OTUs) were clustered with 97% similarity cutoff using UPARSE (version 7.1 http://drive5.com/uparse/), and chimeric sequences were identified and removed using UCHIME. The taxonomy of each 16S rRNA gene sequence was analyzed using RDP Classifier (http://rdp.cme.msu.edu/) against the SILVA (SSU115) 16S rRNA database with a confidence threshold of 70% [31]. One-way ANOVA (SPSS software package) was used to evaluate the soil parameters, plant biomass, plant height, species richness, and root element contents. Pairs of mean values were compared using least significant difference (LSD). Bivariate correlations were used to determine the correlation of species richness with soil pH, litter amount, soil moisture, soil inorganic N concentrations, and AP concentrations. Stepwise multiple linear regressions were used to identify the most important factor affecting species richness after the addition of the mixed materials. All the statistical analyses were performed using SPSS 21.0. P values of less than 0.05 were statistically significant.

## Results

### Effects of the Mixed Materials on Soil Physicochemical Properties

Bulk density, specific gravity, and porosity of soil were determined to investigate the effects of different compositions of the mixed material. Averaged across all sources of variation, porosity dominated the improvement of soil physical structure in the study region, with an increase of 27.28% (16.72%-32.45%), while bulk density and specific gravity decreased by 23.62% (14.34%-29.50%) and 1.61% (0.05%-4.06%), respectively (Table 3). Results of L9(3^4^) orthogonal test showed these effects varied with the composition materials, and the order of influence on the physical properties of low-quality soil is: HA> SAP> B (Table 4). However, the specific gravity didn’t change significantly, and adding a minimum of 3 g·kg^-1^ of HA has the best effect on reducing soil bulk density and increasing porosity, which could meet the conditions of normal plant growth of <1.25 g·cm^-3^ [32]. SAP and B were the best at 3 g·kg^-1^ and 15 g·kg^-1^.

The distributions of soil aggregate sizes are shown in Table 5. The proportion of water stable aggregates larger than 0.25 mm was significantly increased by 14.79%-40.53% in E1-E9 compared with the CK, respectively (*p* = 0.008, *p* < 0.05) (Table 5). The materials significantly increased in agglomerates larger than 1 mm (*p*_>5mm_ = 0.025, *p*_2-5mm_ = 0.037, *p*_1-2mm_ = 0.003, *p* < 0.05). Aggregates larger than 5 mm, 5-2 mm and 1-2 mm increased by 10.00-95.00%, 5.96-90.42%, and 18.52-192.59%. As Table 4 showed, the effects of the three raw materials on increasing the aggregates >0.25 mm in the soil are as follows: SAP>HA>B. Moreover, the best combination to improve soil physical properties is 3 g·kg^-1^ SAP, 3 or 6 g·kg^-1^ HA (indicating that the difference does not exceed 1%) and 15 g·kg^-1^ B (Table 4).

**Fig. 1:**
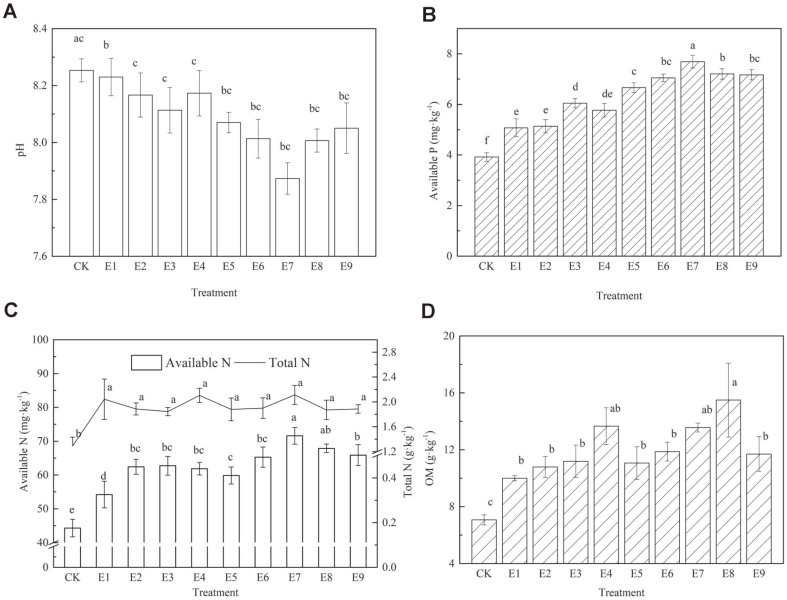
Soil chemical properties treated with mixed materials at different proportions. Different letters indicate significant differences among fertilization treatments based on a one-way ANOVA followed by Tukey’s test (*p* < 0.05).

The chemical properties of the soil samples, including pH, available P, available N, total N, and organic matter are shown in Figs. 1A-1D, respectively. Soil pH was decreased by 0.84%-5.67% compared to CK, and soil available P, total N, available N and organic matter achieved significant increases of 29.34%-97.96%, 42.78%-63.57%, 22.34%-61.50%, and 41.44%-119.94%, respectively (Fig. 1). The results in Table 4 showed that SAP had the greatest effect on available nutrients. Soil organic matter is most affected by B and SAP. In addition, HA has the most significant effect on total N.

In general, the order of influence of mixed materials on soil maturation was SAP > HA >B (Table 4). The optimal combination was SAP_3_HA_3_B_10_, which is consistent with the test results (E7, a mixture of 3 g·kg^-1^ SAP, 3 g·kg^-1^ HA and 10 g·kg^-1^ provides the best improvement in all treatments).

### Soil Bacterial Community Richness and Diversity Analysis

After high-quality trimming and removal of chimeras, 446,425 bacterial sequences were obtained from the mixed materials, and the sequences were clustered into 3,639 bacterial operational taxonomic units (OTUs) with 97% identity as a cutoff (Table S1). All the rarefaction curves began to level off, suggesting that the microbial communities were reasonably characterized by the sampling effort (Fig. S3). The coverage of bacterial communities was higher than 97% for all samples (Table 6). The ACE and Chao 1 results (Table 6) showed that the richness of bacterial communities was higher in E1 than other samples and E9 was lower than other treatments. The results of Shannon and Simpson showed that the highest and lowest bacterial diversity were obtained by E9 and E4, respectively.

### Effects of the Mixed Materials on the Composition of Soil Bacterial Community

**Fig. 2:**
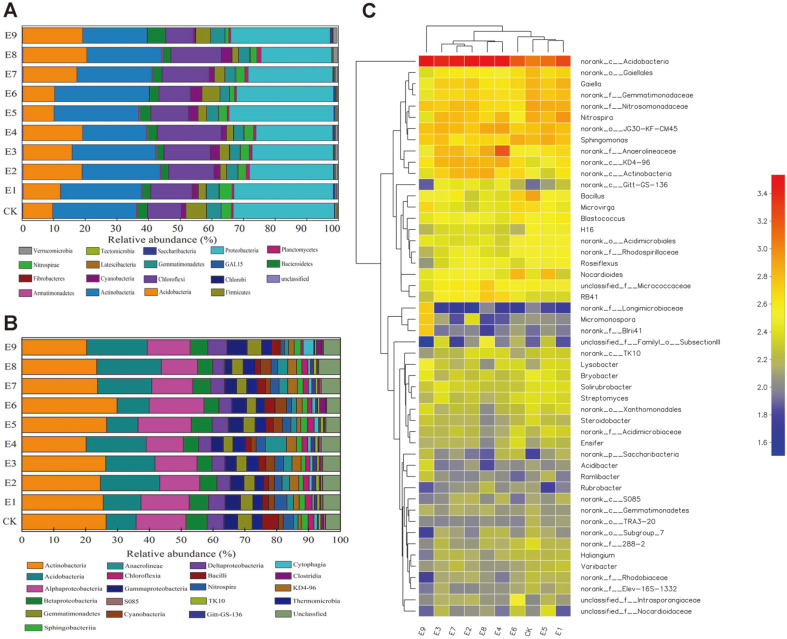
Microbial community structures of all ten samples under different treatments at different classification levels: (A) phylum level, (B) class level (C) cluster analysis and heat map showing the bacterial community composition of each sample based on an analysis of the 50 most abundant genera. The abundance was expressed as a proportion of the total effective sequences in the soil samples.

In this study, bacterial phylum and class with relative abundances above 0.1% of the total community were defined as dominant. The main phyla and classes were found in all samples with different amounts (Figs. 2A and 2B). The dominant 17 bacterial phyla in ten different treatments were Acidobacteria, Actinobacteria, Armatimonadetes, Bacteroidetes, Chlorobi, Chloroflexi, Cyanobacteria, Firmicutes, GAL15, Gemmatimonadetes, Latescibacteria, Nitrospirae, Planctomycetes, Proteobacteria, Saccharibacteria, Tectomicrobia, and Verrucomicrobia. Among them, the most abundance bacterial phyla were Acidobacteria (E8, 20.18%), Actinobacteria (E6, 29.87%), Chloroflexi (E4, 20.23%), and Proteobacteria (E9, 31.40%). The dominant 18 bacterial classes in ten treatments were *Actinobacteria*, *Acidobacteria*, *Alphaprotebacteria*, *Betaproteobacteria*, *Deltaprobacteria*, *Gammaproteobacteria*, *Gemmatimonadetes*, *Thermomicrobia*, *Bacilli*, *Cyanobacteria*, *Nitrospira*, *Anaerolineae*, KD4-96, *Sphingobacteriia*, *Chloroflexia*, *Cytophagia*, and Gitt-GS-136. Among them, the most abundant bacterial classes were Actinobacteria (E6, 29.87%), Acidobacteria (E8, 20.18%), and *Alphaprotebacteria* (E4, 11.72%).

**Fig. 3:**
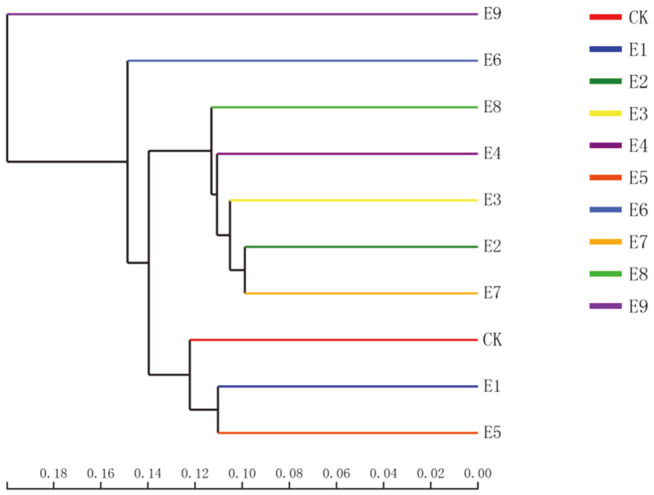
Hierarchical clustering tree of the bacterial community composition at the OTU level based on Bray- Curtis distances.

Bacterial sequences were assigned to a total of 641 recognized and unclassified genera. The heat map showed the profiles of the 50 most abundant genera in samples with different treatments (Fig. 2C). As shown in Fig. 2C, Acidobacteria_norank predominated in all samples, accounting for 4.82-14.53% of the total effective sequences in each sample. Almost all samples contained large amounts of *Nitrospira*, *Sphingomonas*, *Gaiella*, *Bacillus*, *Nocardioides*, *Microvirga*, *Blastococcus*, *Roseiflexus*, *Bryobacter*, *Solirubrobacter*, *Streptomyces*, *Lysobacter*, and*Steroidobacter*. These bacteria played a very good role in soil improvement. *Nitrospira* could oxidize nitrite to nitrate [33]. *Sphingomonas* promoted plant survival in saline-alkali environments and could also produce catalase to promote water retention and regulate soil physical structure [34, 35]. *Bacilli* strains were determined to have nitrogenase and phosphate solubilization activity and produce siderophores, which could prevent the loss of fertilizer [36]. *Streptomyces* provided strong support of nitrogen fixation [37]. The similarity of bacterial community composition among the different treatments at the OTU level was analyzed using clustering analysis (Fig. 3), showing strong similarities between CK, E1, and E5 and between E2, E3, and E7. However, there was no obvious relationship between E9 and other samples.

**Fig. 4:**
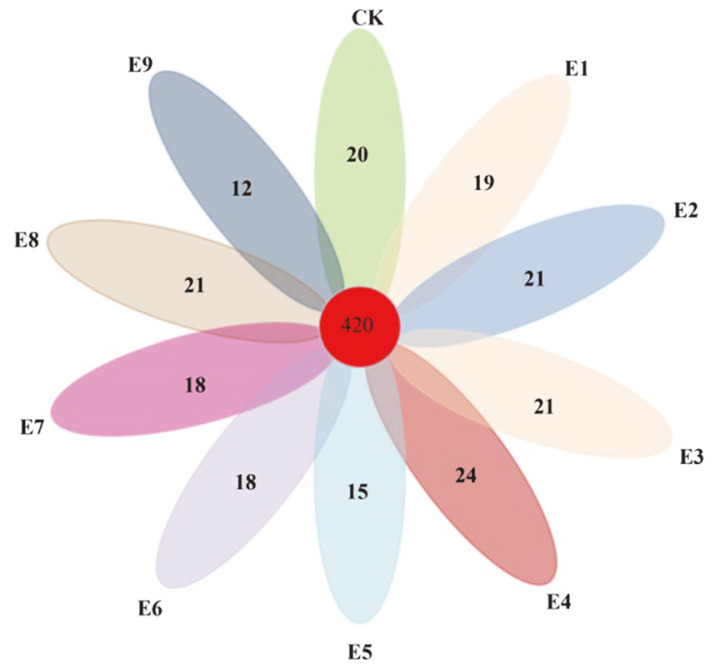
Venn diagram of OTUs at cutoff of 0.03 for bacterial communities. The numbers in the overlapping zones indicate OTUs shared between samples.

Comparison of the bacterial OTUs shared among CK and three environmental materials at nine levels showed that the unique OTUs of different treatments in CK, E1, E2, E3, E4, E5, E6, E7, E8, and E9 were 20, 19, 21, 21, 24, 15, 18, 18, 21, and 12 respectively. The hierarchical clustering analysis showed strong similarities between CK, E1, and E5, and the bacterial communities of E2, E3, and E7. The shared OTUs of the ten samples types were 420, which accounted for 11.54% of the total OTUs (Fig. 4). Among the samples, CK had the most OTUs and E9 had the fewest OTUs

### Effect of the Mixed Materials on Plant Growth

**Fig. 5:**
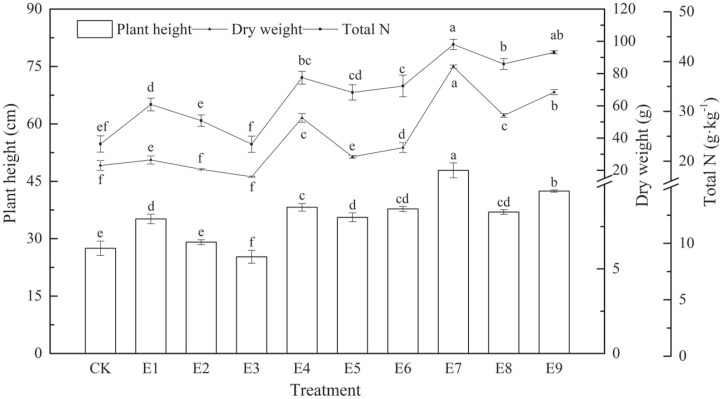
Effects of the mixed materials on the biomass of rye. Different letters indicate significant differences between different treatments based on a one-way ANOVA followed by Tukey’s test (*p* < 0.05).

The important characteristics of plant growth, such as dry weight, plant height and plant total N were measured. Except E3, which decreased by 21.68%, plant dry weight was increased by 28.06%-266.41%, respectively. Significant differences among nine treatments (E1-E9) were observed (*p* = 0.001, *p* < 0.05). Except E3, plant height increased by 5.82%-74.06%, respectively. Significant differences between the mixed materials with different proportions at nine levels were observed (*p* = 0.000, *p* < 0.05). Except E3, plant total N increased by 19.95%- 85.18%, respectively. Significant differences between the mixed materials with different proportions at nine levels were observed (*p* = 0.000, *p* < 0.05). E7 obtained the best performance, with a plant dry weight and plant height of 84.24 g and 44.77 cm, respectively (Fig. 5).

Orthogonal test results of plant height,weight and plant total N, K and R values are displayed in Table 7, and the analysis of variance is shown in Table 8. It has been noted that the combination of SAP3, HA3 and B10 obtained the best performance, which is consistent with the formula of E7. In addition, the analysis of variance showed that SAP was the most important factor for promoting plant growth, and HA was the second. B had non-significant interactions. It could be seen from Table 7 that the influence level of plant height, dry weight and plant total N varied sequentially with the order of SAP > HA > B.

### Relationships between Community Composition and Biochemical Properties

**Fig. 6:**
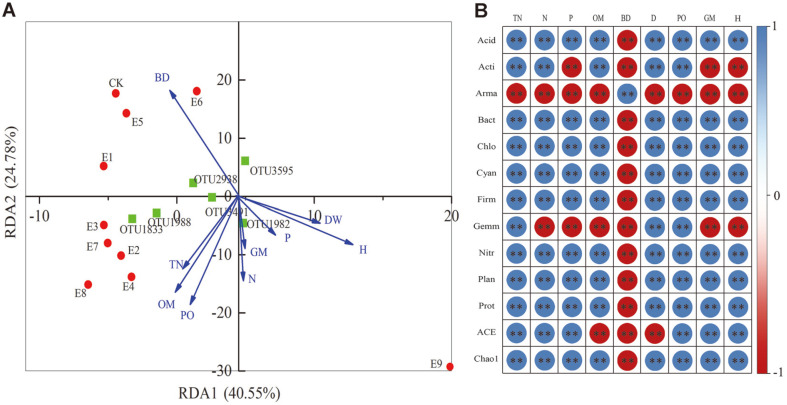
(A) Redundancy analysis (RDA) showing correlations among environmental factors and microbial communities based on OTU for all samples. RDA 1 and 2 explained 40.55% and 24.78% of the total variations, respectively. (B) Pearson’s rank correlation coefficients between microbial community composition, diversity, richness, and soil parameters. Total N (TN), available nitrogen (N), available phosphorus (P), organic matter (OM), bulk density (BD), soil specific gravity (D), porosity (PO), germination (GM), plant height (H). Acidobacteria (Acid), Actinobacteria (Acti), Armatimonadetes (Arma), Bacteroidetes (Bact), Chloroflexi (Chlo), Cyanobacteria (Cyan), Firmicutes (Firm), Gemmatimonadetes (Gemm), Nitrospirae (Nitr), Planctomycetes (Plan), Proteobacteria (Prot), **(*p* < 0.01).

RDA indicated that 63.09% of the total variance within the abundance values of all species was explained by the first (40.55%) and the second (24.78%) ordination axes. The variation in bacterial composition was significantly explained by bulk density, dry weight, plant height, total nitrogen, organic matter, porosity, available nitrogen, and available phosphorus. They were well correlated with the community composition of total bacteria (with the longer arrow) (Fig. 6A). The relationships between soil properties, dominant bacteria phyla, and bacterial diversity and richness were analyzed using Pearson correlation analysis (Fig. 6B). Acidobacteria, Bacteroidetes, Chloroflexi, Cyanobacteria, Firmicutes, Nitrospirae, Planctomycetes, and Proteobacteria were significantly positively correlated with total nitrogen, available nitrogen, available phosphorus, organic matter, soil specific gravity, porosity, germination, and plant height, except for bulk density. Actinobacteria was significantly positively correlated with total nitrogen, available nitrogen, organic matter, soil specific gravity, porosity, while significantly negatively correlated with available phosphorus, germination, and plant height. Armatimonadetes was significantly negatively correlated with total nitrogen, nitrogen, available phosphorus, organic matter, soil specific gravity, porosity, germination, and plant height, except for bulk density. Gemmatimonadetes was significantly positively correlated with total nitrogen, soil specific gravity, porosity, while significantly negatively correlated with available nitrogen, organic matter, bulk soil, germination, and plant height. The ACE and Chao 1 indices were significantly positively correlated with total nitrogen, nitrogen, available phosphorus, porosity, germination, plant height, however significantly negatively correlated with organic matter, bulk density, and soil specific gravity.

## Discussion

### Influence of the Mixed Materials on the Soil Physiochemical Properties

Humic acids were distinguished by the presence of three predominant molecular components: lignin-like molecules, carboxyl-containing aliphatic molecules, and condensed aromatic molecules that were similar to black carbon [38]. Specifically, the super absorbent polymer was bulk-polymerized, crushed crystalline partial sodium salt of cross-linked polypromancic acid with an irregular shape [39]. The biochar had few available nutrients, however it could be beneficial in nitrate loss prevention and carbon sequestration [9]. In this study, diverse effects on available nitrogen were found with the application of the mixed materials to soil. The increases of available nitrogen in soils were obtained by adding the mixed materials. Among different treatments, E7 has the most obvious effect on soil available nitrogen. The reason was that the three materials, especially humic acid, could promote the activity of soil microorganisms and enzymes and accelerate the mineralization of organic matter and the release of nutrient elements.

As shown in Fig. 2, the maximum increase of available P in soil after the addition of the mixed materials was 3 g·kg^-1^. Generally, Ca_3_(PO_4_)_2_ in soil is difficult to dissolve in water, while hydrogen phosphate and dihydrogen phosphate formed by adding humic acid are soluble in water and easily absorbed by crops. After applying humic acid to the soil, a film is formed on the surface of Fe^3+^ and Al^3+^ due to the negative electric properties of humic acid colloid, which can isolate cations from phosphate ions, reduce the chance of insoluble salt formation due to their combination, and improve the relative effectiveness of the applied phosphate fertilizer [7, 40].

Soil bulk density was also significantly affected by the mixed material, consistent with a previous report [41]. Humic acid, combined with physiological effects of plant roots, could accelerate the formation of the soil aggregate structure [42]. Meanwhile, super absorbent polymer could effectively improve the rhizosphere water environment of crops and directly provide preserved water to crops, which can promote the formation of soil aggregate structures, especially the rapid growth of large aggregates [43]. As the soil aggregate structure improved, the bulk density decreased and porosity increased and became permeable. The addition of biochar also caused an increase in organic matter in the soil, which made the soil porous and reduced bulk density. Among the aggregates between 0.25-2 mm, soil nutrients such as total N were mainly present here, and the peroxidase also showed the highest interval activity [44].

### Influence of the Mixed Materials on Soil Microbial Communities

Changes in species richness were found after adding the mixed materials. This result is consistent with the previous literature [45,46]. Additionally, the results in this study indicated that the addition of the mixed materials caused soil acidification, which was positively correlated with species richness. It is possible that certain plant species were not able to adapt to acidic soils due to alterations in soil nutrient balance, resulting in disruption of plant nutrient acquisition.

The results clearly showed that different treatments influenced the soil bacterial community structure. It is well known that additions of different materials have strong influences on soil microbial community structures [47- 49]. The main taxa were present in all treatments after the addition of the mixed material in different amounts (Figs. 2A-2C). For example, Proteobacteria and Actinobacteria existed in all the samples, but more in E9 and E6. The reason was that humic acid can promote the activity of soil microorganisms and enzymes, which accelerated the mineralization of organic matter and promoted nutrient release [50]. The increase in total soil microorganism abundance after biochar addition was because biochar nutrients can promote the transformation of NH_3_ and NH_4_^+^ into NO_3_^-^ in soil. Studies showed that higher temperatures are beneficial for biomass carbon to adsorb NO_3_^-^, thereby reducing the loss of available nitrogen in the soil [51]. Meanwhile, the application of biochar can change soil pH, nutrient availability, and other soil physicochemical properties, indirectly affecting microbial growth and activities, which is consistent with a previous study [52, 53].

The results of the hierarchical clustering analysis also demonstrated the effects of adding the mixed materials on the bacterial community composition at the OTU level.

### Influence of Mixed Materials on Plant Growth

The changes in microbial taxa and compositions can influence plant growth by enhancing nutrient recycling [54]. The application of biochar and humic acid increases microbial growth and activity in soil, which may improve plant performance [55]. Previous research demonstrated that the application of N, P, K, biochar, and humic acid can result in significantly higher acid phosphatase [14]. Previous studies have also reported that available phosphorus plays an important role in plant growth under sufficient nitrogen conditions. It was shown that the proper addition of N enhances C and P accumulation, improving the utilization efficiency and absorption efficiency of phosphorus. Several studies showed that humic acid significantly increased root growth and improved yield in maize [56, 57]. It can also be related to modulation of the microbial community [58]. Other reports indicated that biochar and fertilizer application may reduce nutrient limitation for microbial competition and nutrient leaching, improving plant root biomass and microbial growth [59, 60].

In summary, the mixed material of SAP, HA and B significantly improved the physical and chemical properties of soil. Effects on physical and chemical properties were consistent with the results of plant growth analysis. The influence level of soil and plant properties varies with the order of SAP > HA > B. Orthogonal experiments revealed that the optimal proportion of the mixed material was 3 g·kg^-1^ SAP: 3 g·kg^-1^HA: 10 g·kg^-1^B. The physical and chemical properties caused by different proportions also changed the diversity of soil microorganisms. Acidobacteria, Bacteroidetes, Chloroflexi, Cyanobacteria, Firmicutes, Nitrospirae, Planctomycetes, and Proteobacteria were significantly positively correlated with most of the physical, chemical and plant indicators. Actinobacteria and Armatimonadetes were significantly negatively correlated with most measurement factors. This study will also serve as a valuable reference for the improvement of soil.

## Supplemental Materials



Supplementary data for this paper are available on-line only at http://jmb.or.kr.

## Figures and Tables

**Table 1:** Physicochemical characteristics of the soil.

Category	pH	TN (g·kg^-1^)	TP (g·kg^-1^)	OM (g·kg^-1^)	N (mg·kg^-1^)	P (mg·kg^-1^)	BD	PO
0-20 cm	8.15	0.93	0.17	4.21	57.58	34.28	1.23	0.53
>60 cm	8.21	0.64	0.11	2.87	35.66	22.36	1.41	0.46

Total nitrogen (TN), total phosphorus (TP), organic matter (OM), available nitrogen (N), available phosphorus (P), bulk density (BD), porosity (PO)

**Table 2:** Different fertilization treatments.

Treatment	SAP (g·kg^-1^)	HA (g·kg^-1^)	B (g·kg^-1^)
CK	-	-	-
E1	0.5	3	5
E2	0.5	6	10
E3	0.5	9	15
E4	1	3	15
E5	1	6	5
E6	1	9	10
E7	3	3	10
E8	3	6	15
E9	3	9	5

**Table 3:** Soil physical properties treated with mixed materials at different proportions.

Treatment	Bulk Density (g·cm^-3^)	Specific Gravity (g·cm^-3^)	Porosity (%)
CK	1.44±0.012a	2.60±0.015a	44.77±0.151f
E1	1.07±0.023de	2.59±0.016ab	58.79±0.658a
E2	1.08±0.022cde	2.59±0.007a	58.89±0.751ab
E3	1.12±0.011cd	2.51±0.008bc	55.12±0.313cd
E4	1.06±0.040de	2.60±0.044a	58.36±0.840a
E5	1.15±0.013c	2.53±0.021abc	54.64±0.148d
E6	1.23±0.018b	2.59±0.021ab	52.27±0.343e
E7	1.02±0.009e	2.50±0.026c	59.32±0.053a
E8	1.06±0.050de	2.58±0.034ab	58.96±0.014a
E9	1.11±0.015cd	2.57±0.037abc	56.70±0.028bc

Different letters within the same column indicate significant differences among fertilization treatments based on a one-way ANOVA followed by Tukey’s test (*p* < 0.05).

**Table 4:** Results of L9(3^4^) orthogonal test of soil properties.

Level	Index	Bulk Density	Specific Gravity	Porosity	>0.25μm Aggregates	pH	Total N	Available N	Available P	OM
SAP	K1	3.28	7.69	1.73	71.33	24.51	5.77	179.35	16.26	31.98
	K2	3.45	7.73	1.65	68.60	24.26	5.88	187.01	19.48	36.59
	K3	3.19	7.65	1.75	82.67	23.93	5.87	205.34	22.14	40.75
	R	0.26	0.08	0.10	14.07	0.58	0.11	25.99	5.88	8.77
HA	K1	3.14	7.69	1.76	75.32	24.28	6.26	187.67	18.61	37.22
	K2	3.30	7.71	1.72	76.48	24.24	5.63	190.16	19.01	37.35
	K3	3.47	7.66	1.64	70.81	24.18	5.63	193.87	20.27	34.75
	R	0.33	0.05	0.12	5.67	0.1	0.63	6.20	1.66	2.60
B	K1	3.33	7.69	1.70	73.92	24.35	5.81	179.96	18.91	32.77
	K2	3.34	7.68	1.70	73.94	24.05	5.90	199.26	19.95	36.22
	K3	3.25	7.69	1.72	74.74	24.29	5.82	192.48	19.02	40.34
	R	0.01	0.01	0.02	0.80	0.30	0.90	19.30	1.05	7.57

**Table 5:** Distributions of soil aggregates in different treatments.

Treatment	Soil water-stable aggregates (%)				

	>5 mm	2-5 mm	1-2 mm	0.5-1 mm	0.25-0.5 mm
CK	0.14±0.025b	0.50±0.07b	2.35±0.17c	6.07±0.28b	9.41±0.80b
E1	0.21±0.035b	0.64±0.04b	3.39±0.48b	7.93±0.19b	11.81±0.56a
E2	0.21±0.015b	0.70±0.015b	3.05±0.11bc	8.27±0.18ab	12.98±0.30a
E3	0.20±0.015b	0.55±0.0040b	2.44±0.11c	7.50±0.38b	11.50±0.25a
E4	0.21±0.045b	0.80±0.020ab	2.81±0.24bc	8.79±1.34ab	11.56±0.65a
E5	0.16±0.020b	0.55±0.040b	2.49±0.23c	8.26±0.21ab	11.43±0.67a
E6	0.13±0.025b	0.73±0.24ab	2.74±0.25bc	7.42±1.40b	10.56±1.32a
E7	0.29±0.065ab	0.87±0.020ab	3.82±0.21ab	9.14±1.11ab	13.09±1.85a
E8	0.40±0.025a	0.98±0.045a	4.48±0.34a	9.18±1.09ab	13.39±0.11a
E9	0.30±0.090ab	0.60±0.025a	2.72±0.24bc	10.57±0.33a	12.89±1.14a

Different letters within the same column indicate significant differences among fertilization treatments based on a one-way ANOVA followed by Tukey’s test (*p* < 0.05).

**Table 6:** Different indexes of microbial communities’ richness and diversity in different samples.

Sample	Coverage	ACE	Chao 1	Shannon	Simpson
CK	0.97	2969.87	3031.12	6.78	0.0027
E1	0.98	3232.12	3256.57	6.88	0.0023
E2	0.98	3119.50	3131.36	6.82	0.0026
E3	0.98	3180.68	3152.67	6.84	0.0026
E4	0.98	3176.10	3206.63	6.92	0.0023
E5	0.98	3126.04	3170.21	6.82	0.0025
E6	0.98	3043.62	3069.09	6.65	0.0032
E7	0.98	3143.05	3099.03	6.85	0.0025
E8	0.98	3097.63	3115.19	6.81	0.0029
E9	0.98	2878.43	2914.12	6.51	0.0034

**Table 7:** Results of L9(3^4^) orthogonal test of plant height, dry weight and total nitrogen.

Level	SAP				HA				B			

Index	K1	K2	K3	R	K1	K2	K3	R	K1	K2	K3	R
Plant height	89.53	111.53	127.27	37.73	121.23	101.63	105.47	19.60	113.17	114.73	100.43	14.30
Dry weight	63.05	114.94	206.74	143.70	163.15	103.02	118.56	60.13	123.28	138.93	122.53	16.40
Total N	82.94	105.66	124.81	41.87	111.59	101.45	100.37	11.22	107.05	106.68	99.68	7.37

**Table 8:** Analysis of variance.

Factor	Sum of squares	Freedom	Mean square error	F	Sig
SAP	8281.11	2	4140.55	361.91	a
HA	1623.30	2	811.65	70.94	a
B	200.95	2	100.48	8.782	b
Error	228.818	20	11.441		

F(0.05) = 19 ^a^ *p* < 0.05 ^b^ *p* > 0.05

## References

[1] Feng Y, Wang J, Bai Z, Reading L (2019). Effects of surface coal mining and land reclamation on soil properties: a review. Earth-Sci. Rev..

[2] Bao N, Wu L, Ye B, Yang K, Zhou W (2017). Assessing soil organic matter of reclaimed soil from a large surface coal mine using a field spectroradiometer in laboratory. Geoderma.

[3] Guo A, Zhao Z, Zhang P, Yang Q, Li Y, Wang G (2019). Linkage between soil nutrient and microbial characteristic in an opencast mine, China. Sci. Total Environ..

[4] Liu X, Bai Z, Zhou W, Cao Y, Zhang G (2017). Changes in soil properties in the soil profile after mining and reclamation in an opencast coal mine on the Loess Plateau, China. Ecol. Eng..

[5] Maji D, Misra P, Singh S, Kalra A (2017). Humic acid rich vermicompost promotes plant growth by improving microbial community structure of soil as well as root nodulation and mycorrhizal colonization in the roots of Pisum sativum. Appl. Soil Ecol..

[6] Cao Y, Wang B, Guo H, Xiao H, Wei T (2017). The effect of super absorbent polymers on soil and water conservation on the terraces of the loess plateau. Ecol. Een..

[7] Fernando TN, Ariadurai SA, Disanayaka CK, Kulathunge S, Aruggoda AGB (2017). Development of radiation grafted super absorbent polymers for agricultural applications. Energy Procedia.

[8] Yang L, Yang Y, Chen Z, Guo C, Li S (2014). Influence of super absorbent polymer on soil water retention, seed germination and plant survivals for rocky slopes eco-engineering. Ecol. Eng..

[9] Liu X, Zhang A, Ji C, Joseph S, Bian R, Li L (2013). Biochar's effect on crop productivity and the dependence on experimental conditionsa meta-analysis of literature data. Plant Soil.

[10] Cao X, Ma L, Gao B (2009). Dairy-Manure derived biochar effectively sorbs lead and atrazine. Environ. Sci. Technol..

[11] Lehmann J, Rillig MC, Thies J, Masiello CA, Hockaday WC, Crowley D (2011). Biochar effects on soil biota - a review. Soil Biol. Biochem..

[12] Zhu X, Chen B, Zhu L, Xing B (2017). Effects and mechanisms of biochar-microbe interactions in soil improvement and pollution remediation: a review. Environ. Pollut..

[13] Akmal M, Maqbool Z, Khan KS, Hussain Q, Ijaz SS, Iqbal M (2019). Integrated use of biochar and compost to improve soil microbial activity, nutrient availability, and plant growth in arid soil. Arab. J. Geosci..

[14] Madhavi P, Sailaja V, Ram PT, Hussain SA (2015). Effect of fertilizers, biochar and humic acid on soil enzymes at ferment stages. Int. J. Curr. Res..

[15] Gregorutti VC, Caviglia OP (2019). Impact of crop aerial and root biomass inputs on soil nitrifiers and cellulolytic microorganisms. Soil Till. Res..

[16] Dubey RK, Dubey PK, Abhilash PC (2019). Sustainable soil amendments for improving the soil quality, yield and nutrient content of Brassica juncea (L.) grown in different agroecological zones of eastern Uttar Pradesh, India. Soil Till. Res..

[17] Menon RR, Kumari S, Kumar P, Verma A, Krishnamurthi S, Rameshkumar N (2019). Sphingomonas pokkalii sp. nov., a novel plant associated rhizobacterium isolated from a saline tolerant pokkali rice and its draft genome analysis. Syst. Appl. Microbiol..

[18] Zhou L, Monreal CM, Xu S, McLaughlin NB, Zhang H, Hao G (2019). Effect of bentonite-humic acid application on the improvement of soil structure and maize yield in a sandy soil of a semi-arid region. Geoderma.

[19] Pukalchik M, Mercl F, Terekhova V, Tlustoš P (2018). Biochar, wood ash and humic substances mitigating trace elements stress in contaminated sandy loam soil: evidence from an integrative approach. Chemosphere.

[20] Fan R, Luo J, Yan S, Zhou Y, Zhang Z (2015). Effects of biochar and super absorbent polymer on substrate properties and water spinach growth. Pedosphere.

[21] Xu S, Wang B, Li Y, Jiang D, Zhou Y, Ding A (2020). Ubiquity, diversity, and activity of comammox Nitrospira in agricultural soils. Sci. Total Environ..

[22] Plante AF, Conant RT, Stewart CE, Paustian K, Six J (2006). Impact of soil texture on the distribution of soil organic matter in physical and chemical fractions. Soil. Sci. Soc. AM. J..

[23] Gasmi I, Aljoumani B, Sànchez-Espigares JA, Mechergui M, Moussa M (2019). A linear mixed effect (LME) model for soil nutrients and soil salinity changes based on two localized irrigation techniques (drip irrigation and buried diffuser). Arab. J. Geosci..

[24] Guo A, Zhao Z, Zhang P, Yang Q, Li Y, Wang G (2019). Linkage between soil nutrient and microbial characteristic in an opencast mine, China. Sci. Total Environ..

[25] Six J, Conant RT, Paul EA, Paustian K (2002). Stabilization mechanisms of soil organic matter: implications for C-saturation of soils. Plant Soil.

[26] Tian L, Zhao L, Wu X, Hu G, Fang H, Zhao Y (2019). Variations in soil nutrient availability across Tibetan grassland from the 1980s to 2010s. Geoderma.

[27] Prakash K, Sridharan A, Thejas HK, Swaroop HM (2012). A simplified approach of determining the specific gravity of soil solids. Geotech. Geol. Eng..

[28] Holthusen D, Brandt AA, Reichert JM, Horn R (2018). Soil porosity, permeability and static and dynamic strength parameters under native forest/grassland compared to no-tillage cropping. Soil Till. Res..

[29] Neves CSVJ, Feller C, Guimarães MF, Medina CC, Tavares Filho J, Fortier M (2003). Soil bulk density and porosity of homogeneous morphological units identified by the cropping profile method in clayey oxisols in Brazil. Soil Till. Res..

[30] Amato KR, Yeoman CJ, Kent A, Righini N, Carbonero F, Estrada A (2013). Habitat degradation impacts black howler monkey (*Alouatta pigra*) gastrointestinal microbiomes. ISME J..

[31] Mori H, Maruyama F, Kato H, Toyoda A, Dozono A, Ohtsubo Y (2014). Design and experimental application of a novel non- degenerate universal primer set that amplifies prokaryotic 16S rRNA genes with a low possibility to amplify eukaryotic rRNA genes. DNA Res..

[32] Jorge LC, Cantero M (2003). Soil bulk density and penetration resistance under different tillage and crop management systems and their relationship with barley root growth. Agron. J..

[33] Dahal B, NandaKafle G, Perkins L, Brözel VS (2017). Diversity of free-Living nitrogen fixing Streptomyces in soils of the badlands of South Dakota. Microbiol. Res..

[34] Joshi V, Suyal A, Srivastava A, Srivastava PC (2019). Role of organic amendments in reducing leaching of sulfosulfuron through wheat crop cultivated soil. Emerg. Contam..

[35] Coy RM, Held DW, Kloepper JW (2019). Rhizobacterial colonization of bermudagrass by Bacillus spp. in a Marvyn loamy sand soil. Appl. Soil Ecol..

[36] DeMing J, ChengYou C, Ying Z, ZhenBo C, XiaoShu H (2014). Plantations of native shrub species restore soil microbial diversity in the Horqin Sandy Land, northeastern China. J. Arid. Land.

[37] Volikov AB, Kholodov VA, Kulikova NA, Philippova OI, Ponomarenko SA, Lasareva EV (2016). Silanized humic substances act as hydrophobic modifiers of soil separates inducing formation of water-stable aggregates in soils. CATENA.

[38] Janus A, Pelfrêne A, Heymans S, Deboffe C, Douay F, Waterlot C (2015). Elaboration, characteristics and advantages of biochars for the management of contaminated soils with a specific overview on *Miscanthus* biochars. J Environ. Manage..

[39] Riyazi S, Kevern JT, Mulheron M (2017). Super absorbent polymers (SAPs) as physical air entrainment in cement mortars. Constr. Build. Mater..

[40] Ciarkowska K, Sołek-Podwika K, Filipek-Mazur B, Tabak M (2017). Comparative effects of lignite-derived humic acids and FYM on soil properties and vegetable yield. Geoderma.

[41] Laird DA, Fleming P, Davis DD, Horton R, Wang B, Karlen DL (2010). Impact of biochar amendments on the quality of a typical Midwestern agricultural soil. Geoderma.

[42] Zhang J, Wei Y, Liu J, Yuan J, Liang Y, Ren J (2019). Effects of maize straw and its biochar application on organic and humic carbon in water-stable aggregates of a Mollisol in Northeast China: a five-year field experiment. Soil Till. Res..

[43] Huang W, Liu Z, Zhou C, Yang X (2020). Enhancement of soil ecological self-repair using a polymer composite material. CATENA.

[44] Li Z, Rui Z, Zhang D, Feng X, Lu H, Shen S (2019). Macroaggregates as biochemically functional hotspots in soil matrix: evidence from a rice paddy under long-term fertilization treatments in the Taihu Lake Plain, eastern China. Appl. Soil Ecol..

[45] Bai Y, Wu J, Clark CM, Naeem S, Pan Q, Huang J (2010). Tradeoffs and thresholds in the effects of nitrogen addition on biodiversity and ecosystem functioning: evidence from inner Mongolia Grasslands. Global Change Biol..

[46] Mack MC, Schuur EAG, Bret-Harte MS, Shaver GR, Chapin FS (2004). Ecosystem carbon storage in arctic tundra reduced by long- term nutrient fertilization. Nature.

[47] Chen J, Gong J, Xu M (2020). Implications of continuous and rotational cropping practices on soil bacterial communities in pineapple cultivation. Eur. J. Soil Biol..

[48] Chen C, Zhang J, Lu M, Qin C, Chen Y, Yang L (2016). Microbial communities of an arable soil treated for 8 years with organic and inorganic fertilizers. Biol. Fert. Soils.

[49] Soman C, Li D, Wander MM, Kent AD (2017). Long-term fertilizer and crop-rotation treatments differentially affect soil bacterial community structure. Plant Soil.

[50] Hu J, Wu J, Qu X, Li J (2018). Effects of organic wastes on structural characterizations of humic acid in semiarid soil under plastic mulched drip irrigation. Chemosphere.

[51] Lehmann J, Rillig MC, Thies J, Masiello CA, Hockaday WC, Crowley D (2011). Biochar effects on soil biota - a review. Soil Biol. Biochem..

[52] Gul S, Whalen JK, Thomas BW, Sachdeva V, Deng H (2015). Physico-chemical properties and microbial responses in biochar- amended soils: mechanisms and future directions. Agr. Ecosyst. Environ..

[53] Luo Y, Durenkamp M, De Nobili M, Lin Q, Devonshire BJ, Brookes PC (2013). Microbial biomass growth, following incorporation of biochars produced at 350°C or 700°C, in a silty-clay loam soil of high and low pH. Soil Biol. Biochem..

[54] Zhang Q, Shamsi IH, Xu D, Wang G, Lin X, Jilani G (2012). Chemical fertilizer and organic manure inputs in soil exhibit a vice versa pattern of microbial community structure. Appl. Soil Ecol..

[55] Zhang L, Jing Y, Xiang Y, Zhang R, Lu H (2018). Responses of soil microbial community structure changes and activities to biochar addition: a meta-analysis. Sci. Total Environ..

[56] Wu FZ, Bao WK, Zhou ZQ, Wu N (2009). Carbon accumulation, nitrogen and phosphorus use efficiency of Sophora davidii seedlings in response to nitrogen supply and water stress. J. Arid. Environ..

[57] Canellas LP, Olivares FL, Canellas NOA, Mazzei P, Piccolo A (2019). Humic acids increase the maize seedlings exudation yield. Chem. Biol. Technol. Agric..

[58] Puglisi E, Pascazio S, Suciu N, Cattani I, Fait G, Spaccini R (2013). Rhizosphere microbial diversity as influenced by humic substance amendments and chemical composition of rhizodeposits. J. Geochem. Explor..

[59] Nelissen V, Rütting T, Huygens D, Staelens J, Ruysschaert G, Boeckx P (2012). Maize biochars accelerate short-term soil nitrogen dynamics in a loamy sand soil. Soil Biol. Biochem..

[60] Tian J, Wang J, Dippold M, Gao Y, Blagodatskaya E, Kuzyakov Y (2016). Biochar affects soil organic matter cycling and microbial functions but does not alter microbial community structure in a paddy soil. Sci Total Environ..

