# Supplementary material for: Responses of Low-Quality Soil Microbial Community Structure and Activities to Application of a Mixed Material of Humic Acid, Biochar, and Super Absorbent Polymer
Source: J Microbiol Biotechnol. 2020 Jul 15;30(9):1310–20. doi: 10.4014/jmb.2003.03047 (PMC9728209; doi:10.4014/jmb.2003.03047)
Supplement: Supplementary file 1 [file JMB-30-9-1310-supple.pdf]

J. Microbiol. Biotechnol.
https://doi.org/10.4014/jmb.2003.03047

Table S1. Effective bacterial sequences of different samples.

Sample	CK	E1	E2	E3	E4	E5	E6	E7	E8	E9
Sequences	38518	43860	43285	47033	41596	45467	49413	41858	44728	50667

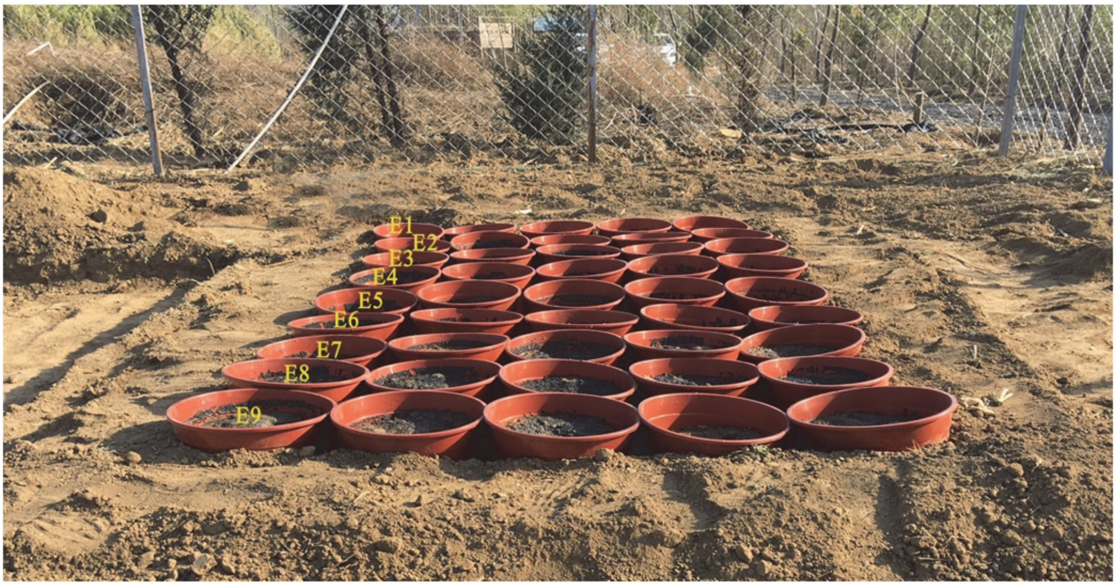

Fig. S1. The process of sowing.

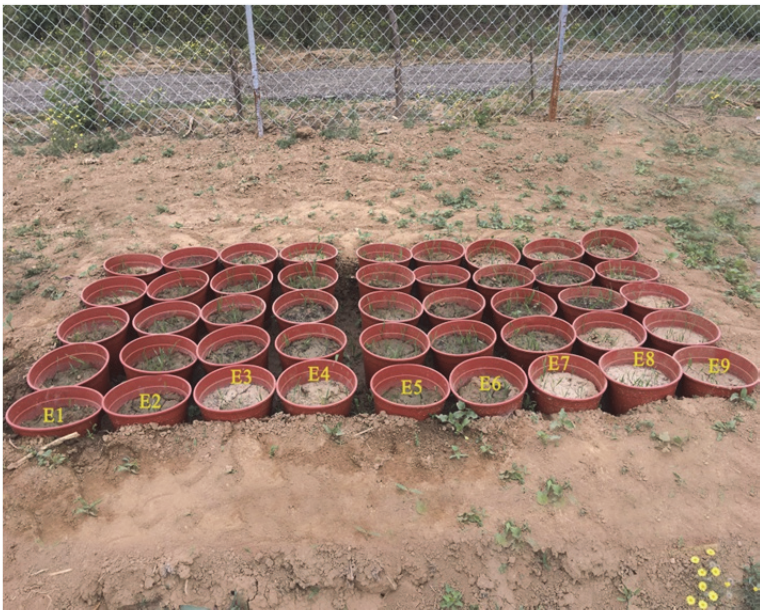

Fig. S2. Plant growth process.

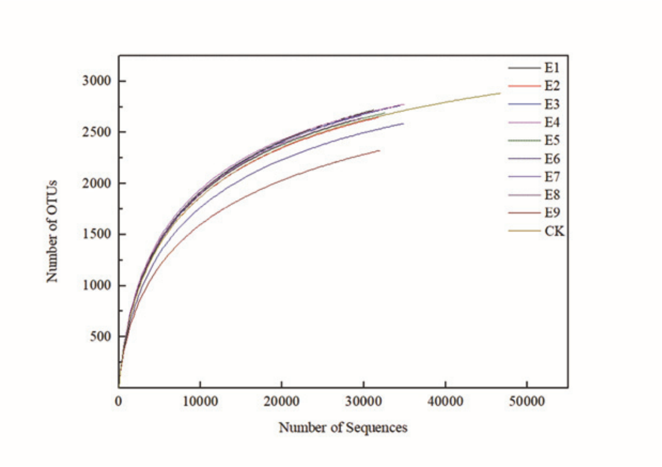

Fig. S3. The rarefaction curve of bacterial.
